# Polymorphisms of CYP2C8 Alter First-Electron Transfer Kinetics and Increase Catalytic Uncoupling

**DOI:** 10.3390/ijms20184626

**Published:** 2019-09-18

**Authors:** William R. Arnold, Susan Zelasko, Daryl D. Meling, Kimberly Sam, Aditi Das

**Affiliations:** 1 Department of Biochemistry, University of Illinois Urbana-Champaign, 3813 Veterinary Medicine Basic Sciences Building, 2001 South Lincoln Avenue, Urbana, IL 61802, USA; william.arnold@ucsf.edu (W.R.A.); susan.e.zelasko@gmail.com (S.Z.); dmeling@illinois.edu (D.D.M.); kimberlytsam@gmail.com (K.S.); 2 Department of Comparative Biosciences, University of Illinois Urbana-Champaign, 3813 Veterinary Medicine Basic Sciences Building, 2001 South Lincoln Avenue, Urbana, IL 61802, USA; 3 Department of Bioengineering, University of Illinois Urbana-Champaign, Beckman Institute for Advanced Science and Technology, 3813 Veterinary Medicine Basic Sciences Building, 2001 South Lincoln Avenue, Urbana, IL 61802, USA; 4 Division of Nutritional Sciences, University of Illinois Urbana-Champaign, 3813 Veterinary Medicine Basic Sciences Building, 2001 South Lincoln Avenue, Urbana, IL 61802, USA

**Keywords:** CYP2C8, polymorphisms, reactive oxygen species, paclitaxel, cytochrome P450 reductase, electron transfer

## Abstract

Cytochrome P450 2C8 (CYP2C8) epoxygenase is responsible for the metabolism of over 60 clinically relevant drugs, notably the anticancer drug Taxol (paclitaxel, PAC). Specifically, there are naturally occurring polymorphisms, CYP2C8*2 and CYP2C8*3, that display altered PAC hydroxylation rates despite these mutations not being located in the active site. Herein, we demonstrate that these polymorphisms result in a greater uncoupling of PAC metabolism by increasing the amount of hydrogen peroxide formed per PAC turnover. Anaerobic stopped-flow measurements determined that these polymorphisms have altered first electron transfer kinetics, compared to CYP2C8*1 (wildtype), that suggest electron transfer from cytochrome P450 reductase (CPR) is disfavored. Therefore, these data demonstrate that these polymorphisms affect the catalytic cycle of CYP2C8 and suggest that redox interactions with CPR are disrupted.

## 1. Introduction

Cytochrome P450 2C8 (CYP2C8) is a member of the cytochrome P450 (CYP) epoxygenase family that metabolizes over 60 clinically relevant drugs on the market [1,2,3]. For example, CYP2C8 is the primary enzyme involved in the metabolism of paclitaxel (PAC), a common chemotherapeutic that works by interfering with microtubule function [4]. CYP2C8 is primarily expressed hepatically, though it is also present in the vasculature and kidneys [5,6,7], where it metabolizes lipids, such as arachidonic acid (AA), to form biologically active epoxyeicosatrienoic acids (EETs). EETs are known to be anti-inflammatory [8], angiogenic [9], and inhibit vascular smooth muscle cell migration, implicating CYP2C8 in regulating kidney and vascular function [10].

Polymorphic variations in CYPs have been of clinical interest due to individual differences in drug metabolism. For example, CYP2D6 is among the most highly polymorphic CYPs that greatly contributes to the poor, intermediate, extensive, and ultra-rapid metabolizer phenotypes [11,12]. Two common, naturally occurring polymorphic variants of CYP2C8—CYP2C8*2 and CYP2C8*3—display altered drug elimination rates and EET production compared to CYP2C8*1 (wildtype, WT) [13,14]. The CYP2C8*3 polymorphism (R139K/K399R) is present in 2% of African-American and 13% of Caucasian populations [13]. CYP2C8*3 is associated with an increase in peripheral neuropathy in patients treated with PAC, presumably a result of slower PAC elimination by CYP2C8*3 [15,16]. However, some studies have suggested PAC metabolism is increased with CYP2C8*3 [17]. In vitro studies corroborate that CYP2C8*3 has only 30% and 15% of the activity compared to WT for the turnover of AA and PAC, respectively [13]. However, others report that PAC metabolism is not significantly affected by these polymorphisms [14,18], and one study observed greater PAC turnover by *3 as compared to WT [19]. Therefore, from previous reports, the effects of CYP2C8*3 on PAC metabolism are convoluted. The CYP2C8*2 (I269F) polymorphism, present in 18% of African-American populations [13], shows lower, albeit not always statistically significant, in vitro PAC turnover compared to WT [13,14,20].

**Figure 1:**
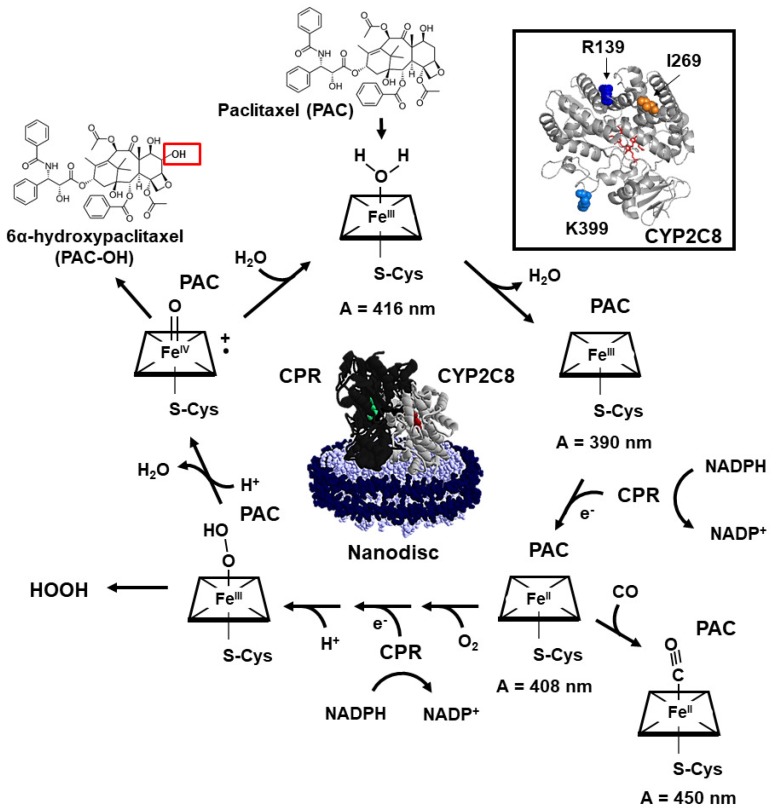
Schematic of the CYP catalytic cycle. Inset shows the structure of CYP2C8 with residues R139 (blue), I269 (orange), and K399 (light blue) highlighted. Structure was generated in PyMol v1.3r1 using the PDB entry 1PQ2. A schematic of CYP2C8 (grey) and CPR (black) incorporated into nanodiscs is shown in the center of the cycles. *Catalytic cycle*. Substrate (PAC) binds to the CYP active site, which perturbs the H_2_O coordination to the iron heme. H_2_O unbinds leaving a pentacoordinated high-spin iron heme. CPR reduces the iron heme using an electron obtained from NADPH. Under anaerobic conditions, CO ligates the heme to terminate the cycle. Under aerobic conditions, O_2_ ligates the heme, followed by another one-electron reduction by CPR and the addition of a proton to produce a peroxy-heme. The peroxy-heme can decompose forming HOOH or eliminate an H_2_O molecule to produce the catalytic ferryl iron heme radical (Compound I). Compound I can oxidize substrate (PAC) to product (PAC-OH, red box), followed by the coordination of an H_2_O molecule to begin the cycle again. Spectroscopically visible species are indicated with their characteristic absorbance wavelength. More details of the cycle can be found in previous reviews [22,23,24,25].

Importantly, the amino acid residues that are different in the CYP2C8*2 and CYP2C8*3 (R139, K399, and I269) are not located within the enzyme active site of CYP2C8 but on the surface of the protein (Figure 1) [2,13,18,19]. This suggests that these mutations disrupt mechanisms of substrate metabolism that are not directly related to substrate binding. Indeed, CYP2C8*3 showed WT activity for the deethylation of amiodarone, and so it appears that this polymorphism does not affect substrate binding as a whole [21]. The CYP catalytic cycle is a complex series of redox reactions that require assistance from redox partners such as cytochrome P450 reductase (CPR) (Figure 1). The details of this complex cycle can be found in many reviews [22,23,24,25]. Therefore, these mutations may be affecting other steps in the CYP catalytic cycle, such as electron transfer from redox partners. Earlier work demonstrated that CYP2C8*3 has different binding affinities to its redox partners compared to WT. Particularly, CPR interacts with PAC-bound CYP2C8*3 better than WT as determined indirectly by PAC metabolism assays using varying CPR concentrations (apparent K_m_ = 5.5 ± 1.5 nM for CYP2C8*3 versus 35 ± 10 nM for WT) [19]. The greater apparent interaction with CPR ought to lead to a faster transfer of electrons and a greater substrate turnover. In the same study, the authors determined that PAC metabolism was increased with CYP2C8*3 compared to WT [19]. However, the consensus through other studies is that PAC metabolism is either lower or similar to WT [13,14,18]. Taken together, these data suggest that the CYP-CPR interaction may be disrupted in CYP2C8*3 and affects PAC metabolism.

CPR transfers two electrons to CYPs during the CYP catalytic cycle, with the first electron reducing the ferric heme to a ferrous heme and the second reducing the dioxygen-heme to a peroxy-heme (Figure 1). Many reactive oxidized intermediates are involved in the CYP catalytic cycle en route to substrate oxidation. These intermediates can sometimes decompose to form reactive oxygen species (ROS) instead of progressing towards substrate oxidation, a process known as uncoupling. These ROS, namely hydrogen peroxide (HOOH) and superoxide, are formed in large quantities by CYP2C enzymes [26,27]. ROS can induce mitochondrial dysfunction in cardiomyocytes, induce coronary artery vasoconstriction, and promote carcinogenesis [26,28,29,30], and ROS specifically generated by endothelial CYP2C8 has been shown to impair functional recovery after ischemia/reperfusion injury [27]. Another potential effect of these polymorphisms may therefore be on the coupling efficiency of PAC metabolism.

Herein, we determined the effects of the CYP2C8*2 and CYP2C8*3 polymorphisms in regards to first-electron transfer (FET) kinetics and PAC metabolism uncoupling. We tested CYP2C8*1, CYP2C8*2, and CYP2C8*3, as well as the single mutations of the CYP2C8*3 polymorphism (R139K and K399R). To study these polymorphisms, we utilized the Nanodisc technology to solubilize these CYP2C8 variants in a membrane mimic [31,32]. We find that CYP2C8*2 has a lower PAC turnover rate compared to WT. We further determined that CYP2C8*3 produces significantly more HOOH compared to WT, indicating a greater uncoupling of the catalytic cycle. Using stopped-flow measurements of the FET, we determined that the CYP2C8*2 and CYP2C8*3 have profoundly distinct and slower FET kinetics compared to WT. We determined that there is no change in the reduction potential of the polymorphisms compared to WT, which supports that the altered FET kinetics is due to an altered redox interaction with CPR. None of the single-mutant variants of CYP2C8*3 (R139K and K399R) reproduce the effects of the CYP2C8*3, indicating that the effects of this polymorphism are a synergism of both mutations. Taken together, these data demonstrate that these polymorphisms have altered FET kinetics, leading to an increase in HOOH production and greater PAC metabolism uncoupling.

## 2. Results and Discussion

### 2.1. P450 Characterization of CYP2C8*1/*2/*3/R139/K399-ND

**Figure 2:**
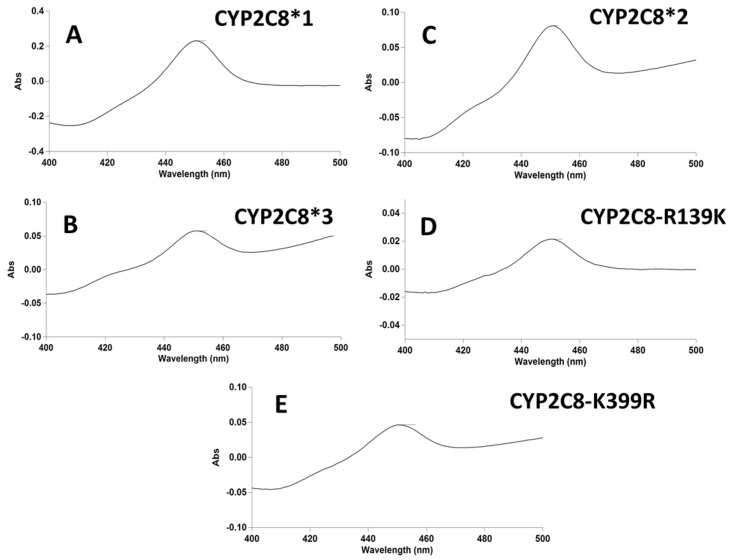
*CO-binding difference spectra.* (**A**) CYP2C8*1, (**B**) CYP2C8*2, (**C**) CYP2C8*3, (**D**) CYP2C8-R139K, and (**E**) CYP2C8-K399R.

In order to assess if these variants of CYP2C8 lead to unfolding of the protein and improper ligation of the heme group, we performed anaerobic CO-binding assays. All the variants showed a 90%–100% shift in the heme absorbance to 450 nm and resemble CYP2C8*1 characteristics (Figure 2 and Table S2). Therefore, these variants are well-folded.

### 2.2. Effect of Polymorphisms on PAC Metabolism

Next, we sought to examine the effect that these polymorphisms have on PAC turnover rates. Previous studies reported a poor solubility of PAC that precluded *V_max_* determination for in vitro CYP2C8 [33], and so we analyzed our data based on the time linearity. Linearity was established for the hydroxylation of 70 µM PAC to 6α-hydroxypaclitaxel (PAC-OH) over a 20 min period. We can estimate the catalytic efficiency of the PAC metabolism among the variants by fitting the data to Equation (1)

(1) $$\left[S\right]=\left[S_{0}\right]e^{-kt}$$

where $\left[S_{0}\right]$ is the initial concentration of the substrate and $k=\frac{k_{cat}}{K_{m}}\left[E\right]$ (Figure S1) [34]. The catalytic efficiencies of these variants range from 0.207 to 1.02 min^−1^ nM^−1^ (Figure S1, Table 1), which are lower than previously reported rates for CYP2C8-mediated in vitro PAC metabolism using a lipid-reconstituted system [33]. CYP2C8*2 showed a marked decrease in turnover rate (47.2% WT), consistent with earlier reports of its inefficient PAC metabolism [13,14]. Compared to WT, CYP2C8*3 had slightly, albeit not significantly, lower PAC turnover rates. These results for CYP2C8*3 agree with the findings of Yu et al. [14] and Soyama et al. [18]. However, they contradict the findings of Dai et al. [13], who were unable to measure PAC metabolism, and contradict the findings of Kaspera et al., who reported higher PAC metabolism [19]. The CYP2C8-K399R variant metabolized PAC with rates similar to WT, but interestingly the CYP2C8-R139K variant showed a remarkable increase in PAC turnover (265% compared to WT). Therefore, the effects of the CYP2C8*3 single mutations do not additively contribute to the CYP2C8*3 activity. Overall, we observed that the CYP2C8*3 polymorphism does not significantly affect PAC turnover, but CYP2C8*2 is half as efficient as WT. Since these polymorphisms do not occur in the active site of CYP2C8, it is unlikely that they directly affect PAC binding. Therefore, we further probed other steps of the CYP catalytic cycle to determine the mechanism through which these polymorphisms affect CYP2C8 activity.

### 2.3. Polymorphisms in CYP2C8 Lead to Greater HOOH Uncoupling

**Figure 3:**
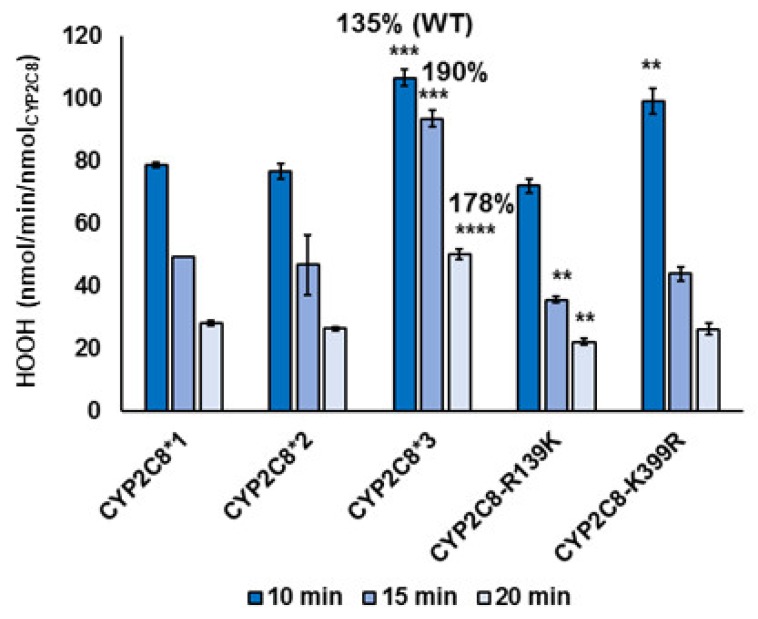
HOOH production rates. The rate of HOOH production by each CYP2C8 variant was measured using an Amplex Red peroxidase kit at 10, 15, and 20 min reaction times, in the presence of 70 μM PAC. Error represents the SEM of 3–4 experiments. Statistical significance was determined by comparing experiments to their respective WT controls. ** *p* < 0.01; *** *p* = 0.0001; **** *p* < 0.0001.

To assess the uncoupling efficiency of PAC metabolism, we next measured the rate of HOOH production over time by each variant. None of the variants showed a significant difference in the HOOH production rates in the presence of 70 µM PAC compared to without. This is likely due to the high amounts of HOOH produced compared to PAC turnover (Figure S2). The overall amount of HOOH linearly decreased for all CYP2C8 variants over time, indicating a burst of activity at the initiation of the reaction followed by decomposition of HOOH (Figure 3). For all time points, CYP2C8*3 displayed nearly 200% higher HOOH production compared to WT. Therefore, the CYP2C8*3 polymorphism leads to a greater ROS production and catalytic uncoupling. PAC and ROS have both been implicated in the pathogenesis of neuropathic pain [35,36], and therefore it would be interesting to see if the increase in HOOH production contributes to the neuropathy observed with CYP2C8*3 [15,16]. There was an increase in HOOH production with the CYP2C8-K399R variant at the start of the reaction and a minor decrease in HOOH production over time with the CYP2C8-R139K variant. Therefore, likewise to the PAC metabolism experiments, the individual mutations of the CYP2C8*3 do not additively contribute to the CYP2C8*3 phenotype. CYP2C8*2 did not have a significant difference in the HOOH production compared to WT, but this polymorphism also displayed half the PAC turnover as WT. By normalizing the amount of HOOH produced to the activity of the enzyme by looking at the HOOH produced per PAC turnover, we see that both CYP2C8*2 and CYP2C8*3 produce almost 200% more HOOH per PAC turnover than WT (Table 2). The CYP2C8*2 and CYP2C8*3 polymorphisms, therefore, are about twofold more uncoupled (i.e., produce twofold more ROS), with CYP2C8*3 producing significantly more HOOH than WT. ROS uncoupling can be caused by altered redox kinetics during the CYP2C8 catalytic cycle or electron transfer from CPR to CYP2C8 (Figure 1). Therefore, we next proceeded to determine if these polymorphisms alter the intrinsic redox potential of the CY2C8 heme.

### 2.4. Spectral Characterization of Substrate Binding and Reduction Potentials of CYP2C8 Polymorphisms

**Figure 4:**
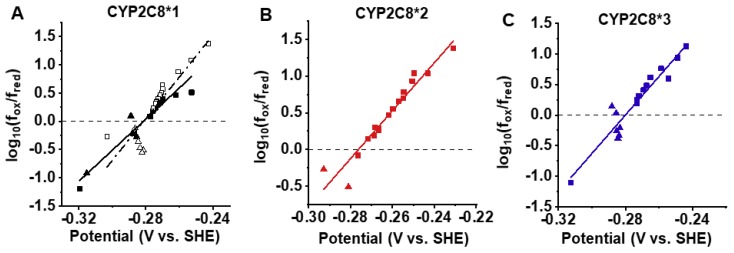
Reduction potential of CYP2C8 variants. Redox titration of CYP2C8 variants was conducted in 0.1 M phosphate buffer (pH 7.4 at 25 °C) with or without 70 μM PAC. The potential was measured spectroscopically by using a safranin T as the redox indicator. Reduction was achieved using light and dithionite and oxidation was achieved using K_3_[Fe(CN)_6_)] as stated in the Methods section. Representative Nernst plots for (**A**) CYP2C8*1 without paclitaxel (open black) and with paclitaxel (solid black), (**B**) CYP2C8*2, and (**C**) CYP2C8*3 from two experiments are shown. Data obtained from reduction is given as squares and data from oxidation is given as triangles. The zero intercept gives *E*°′, the redox potential of the protein.

We determined the reduction potential of the CYP2C8 variants using safranin T as a redox indicator as previously described [37]. Substrates binding to CYPs, such as CYP3A4, often perturbs water binding at the 6th coordinate position to produce a pentacoordinated high-spin heme (Figure 1). This results in an increase in the reduction potential of the heme and helps facilitate electron transfer from CPR [38]. The high-spin content can be observed as a shift in the heme absorbance from ~417 to ~390 nm. We did not observe a significant high-spin shift upon PAC binding to CYP2C8 in any of the variants, as was similarly observed for PUFAs binding to CYP2J2 [39]. We also determined that there is not a significant change in the reduction potential of CYP2C8*1 upon PAC binding (Figure 4A, Table 3), which correlates to the poor high-spin content of the PAC-bound protein. The poor high-spin content and minimal change to the reduction potential together support the slow metabolism of PAC by CYP2C8 compared to other CYP-mediated drug metabolisms. Compared to the polymorphisms, CYP2C8*2 had a slightly albeit not significantly lower reduction potential, and the reduction potential of CYP2C8*3 was similar to WT (Figure 4, Table 3). Altogether, there is not a significant change to the intrinsic redox properties of the heme in these polymorphisms. We next proceeded to measure the first-electron transfer (FET) kinetics between CPR and CYP2C8.

### 2.5. Polymorphisms Show Altered First Electron Transfer (FET) Kinetics as Determined by CO Stopped-Flow

To further probe the effect of these polymorphisms on the CYP2C8 catalytic mechanism, we determined the kinetics of the FET from CPR to the CYP2C8 variants during the metabolism of 70 µM PAC. We firstly determined that there is not a significant change in the NADPH oxidation rates among the variants (Figure S3), which supports previous findings [19]. As NADPH is the initial step in the reaction, we then proceeded to conduct stopped-flow measurements in order to determine how CPR transfers electrons to the CYP2C8 variants.

**Figure 5:**
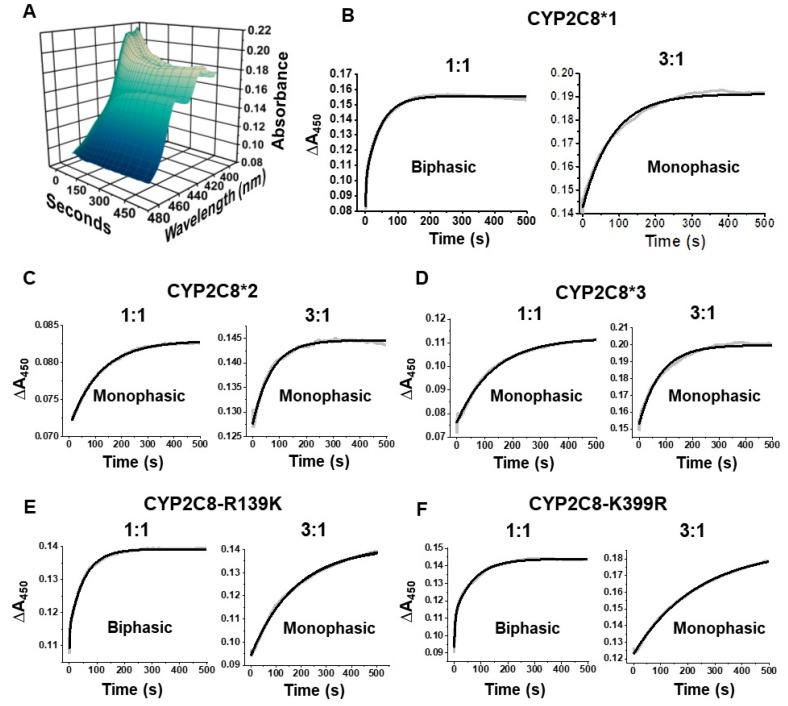
Stopped-flow electron transfer rate to CYP2C8 variants. (**A**) Three-dimensional plot of representative absorbance spectra from 0 to 500 s showing the increase in absorbance near 450 nm following the mixing of NADPH with a pre-incubated complex of paclitaxel-bound CYP2C8 and CPR. (**B**–**F**) Representative plots of the change in peak absorbance at 450 nm from 0 s to 500 s. Experiments were conducted at either a 1:1 CPR:CYP or a 3:1 CPR:CYP ratio. Data points (grey squares) are overlaid with the line of best fit (solid black line) derived from a fit of the data to either monophasic (single exponential) or biphasic (two exponential) kinetic equations in OriginLab as indicated.

CYPs display a characteristic shift in the heme absorbance to 450 nm upon CO binding to the heme. In order for CO to bind, the heme must be reduced to the ferrous state by CPR, which we determine by the appearance of the 450 nm absorbance band over time (Figure 5A). Therefore, the rate at which CO binds to the heme is directly related to the FET rate. The rate of CO binding was monophasic across all variants (Figure 5B–F). Compared to WT, CYP2C8*2 and CYP2C8*3 showed higher rates of CO binding, which may also explain the greater HOOH uncoupling and/or the higher levels of HOOH production by CYP2C8*3. Contrariwise, the CYP2C8-R139K and CYP2C8-K399R variants showed lower rates compared to WT (Table 4).

CYP metabolism studies are typically performed using a saturating 3:1 CPR:CYP ratio in order to achieve a maximum pseudo-zero-order electron transport kinetics between CPR and the CYP. Hitherto, we have used a 3:1 CPR:CYP ratio in our experiments. To determine if the FET is dependent on the interactions of CYP2C8 with CPR, we repeated the experiments by lowering the CPR:CYP ratio from 3:1 to 1:1. CYP2C8*1, CYP2C8-R139K, and CYP2C8-K399R all showed biphasic CO binding at the 1:1 CPR:CYP ratio and had similar fast (k_1_) and slow (k_2_) kinetics compared (Figure 5B,E,F, Table 4). Altering the CPR:CYP ratio has previously been shown to change the number of phases of the FET in certain situations likely by forcing CPR to associate with CYPs in unproductive confirmations at higher CPR:CYP ratios [40]. Likewise, the 3:1 ratio with CYP2C8*1, CYP2C8-R139K, and CYP2C8-K399R may be promoting unproductive confirmations with CPR to produce these observations.

Interestingly, CYP2C8*2 and CYP2C8*3 only show the slow phase of the CO binding at the 1:1 ratio (Figure 5C,D, Table 4). The values of k_2_ for these polymorphisms are about half that compared to WT. However, the FET rate of CYP2C8*2 and CYP2C8*3 rates in the 3:1 CPR:CYP experiments resemble those of the WT slow phase in the 1:1 ratio. Therefore, it appears that more CPR is required for the CYP2C8*2 and CYP2C8*3 polymorphisms to rescue the WT activity of this slow phase. Together, these data suggest that these polymorphisms reduce either the binding of CPR to CYP2C8 or the transfer of electrons from CPR to CYP2C8. We will refer to these two events as the CYP–CPR redox interaction. Kaspera, et al. had previously determined that the apparent affinity of CPR for CYP2C8*3 is greater than WT; however, the study also used a different recombinant system with cytochrome b5 as an auxiliary redox partner [19]. Homology modeling (Figure S4) reveals that R139, K399, and I269 all lie on the putative CYP-CPR binding interface, which means these mutations may be directly interfering with the CYP–CPR interaction. Further, K399 is located near the N-terminus at the protein-membrane interface (Figure 1A), and we showed previously that the N-terminus is essential for the CPR-mediated reduction of CYP2J2, which has a close homology to CYP2C8 [41].

## 3. Conclusions

CYP2C8*2 and CYP2C8*3 contain mutations that are not in the active site of CYP2C8. These mutations are instead located on the periphery of the protein. Therefore, these residues do not directly contribute to substrate binding and instead must affect CYP2C8 activity through their modulation of other steps in the CYP catalytic cycle. These mutations lie on the putative CYP–CPR interface and thus may be affecting the redox interactions between these proteins. We determined that CYP2C8*2 is 47% as active towards PAC turnover compared to WT and that CYP2C8*3 shows WT activity. The metabolism of PAC is 200% more uncoupled in the CYP2C8*2 and CYP2C8*3 polymorphisms, and CYP2C8*3 produces significantly more HOOH compared to WT. Stopped-flow kinetics of the FET suggest that the polymorphisms reduce the transfer of electrons by CPR to CYP2C8. In conclusion, these in vitro studies demonstrate that these polymorphisms of CYP2C8 do not directly affect PAC binding to CYP2C8 but may, in fact, be affecting the redox interaction between CYP2C8 and CPR. The CYP2C8*2 and CYP2C8*3 polymorphisms reduce the CYP–CPR redox interaction and promote greater uncoupling of PAC metabolism. Therefore, not only is the FET disrupted in these polymorphisms, the electrons are being utilized towards ROS formation in lieu of PAC turnover. However, we were unable to definitively determine how the redox interaction is being altered, i.e., whether the polymorphisms affect the ability of CPR to bind and dock to CYP2C8, how the electrons are shuttled through CYP2C8, or both.

Another important finding is that the effects of the CYP2C8*3 polymorphism on CYP2C8 activity cannot be explained by the additive contribution of the individual mutations themselves. In all experiments, CYP2C8*3 had distinct activities compared to the linear combination of the R139K and K399R individual data. In fact, the single mutations either showed WT activity (especially concerning FET kinetics) or were significantly different than either WT or CYP2C8*3 (e.g., the 265% increase in PAC metabolism by CYP2C8-R139K). Therefore, the mutation of these two residues do not additively contribute to the CYP2C8*3 phenotype.

There are many mechanisms by which these polymorphisms effect the CYP2C8 redox interaction. They may be directly affecting the binding of CPR to CYP2C8. The mutations may also be altering the architecture of CYP2C8 such that it disfavors redox interactions with CPR while also destabilizing the peroxy-heme intermediate to produce HOOH. CYP2C8*2 contains an I269F mutation, which is a significant change to the physical and chemical properties of the residue. It is possible that this mutation has a profound effect on the folding of CYP2C8 and the interaction with CPR. CYP2C8*3 contains a R139K/K399R double mutation, which interestingly swaps the Lys and Arg residues. While Lys and Arg are both positively charged residues, they differ significantly in their physical properties. For instance, Arg can form a greater number of electrostatic interactions and better maintains a positive charge compared to Lys. It has been shown that Arg substitution can increase the stability of GFP, which was shown in silico to be facilitated by a greater number of salt bridge interactions [42]. Arg also interacts differently to phospholipids and increases interfacial binding and perturbations to membranes [43]. Therefore, this polymorphism may be affecting how CYP2C8 associates with lipids as well as how it interacts with CPR.

## 4. Materials and Methods

### 4.1. Materials

The human CYP2C8 gene cloned into the Amp^R^ pAr5 (modified pCWOri+) plasmid was a gift from Dr. Eric Johnson. PCR reagents were purchased from New England Biolabs (Ipswich, MA, USA). Molecular biology enzymes and *E. coli* DH5α were purchased from Invitrogen (Waltham, MA, USA). Plasmid DNA was purified using a Qiagen Gel Extraction kit. Ampicillin (Amp), arabinose, chloramphenicol (Chlr), isopropyl β-D-1-thiogalactopyranoside (IPTG), and Ni-NTA resin were purchased from Gold Biotechnology (St. Louis, MO, USA). δ-Aminolevulinic acid (δ-ALA) was purchased from Frontier Scientific (Emeryville, CA, USA). 1-palmitoyl-2-oleoyl-sn-glycero-3-phosphocholine (POPC) and 1-palmitoyl-2-oleoyl-sn-glycero-3-phospho-L-serine (POPS) were purchased from Avanti Polar Lipids (Alabaster, AL, USA). Carbamazepine and paclitaxel were purchased from Cayman Chemicals (Ann Arbor, MI, USA). NADPH was purchased from P212121 Store.

### 4.2. Protein Engineering of CYP2C8*1/*2/*3/R139K/K399R

The CYP2C8 plasmid from Dr. Johnson was used directly for engineering the CYP2C8 variants. Plasmids were amplified and purified using a Qiagen Plasmid mini-prep kit (Valencia, CA, USA). The R139K and K399R single nucleotide substitutions were made using forward and reverse primers containing each mutation and an inserted BspQI restriction enzyme site (New England Biolabs) (Table S1). BspQI is a class II restriction enzyme that will create a unique sticky-end cut one nucleotide removed from the restriction enzyme site. The resulting gene of the R139K amplification and the K399R primers were then used to construct the CYP2C8*3 gene. A single *2 mutation was made similarly containing a single substitution at I269F. The PCR reaction consisted of 1 µM of forward and reverse primers in HF reaction buffer (New England Biolabs) containing 50 pg/µL CYP2C8-containg plasmid, 200 µM dNTPs, 5% DMSO, and Phusion DNA polymerase (10 U/mL). The PCR thermocycler was set to 95 °C for 3 min, 20 cycles (95 °C for 30 sec, 65 °C for 30 sec), and then 72 °C for 4 min. The mutated plasmids were then digested with BspQI and the resulting sticky ends were ligated to make complete plasmids. Chemically competent DH5α cells were transformed by heat shock at 42 °C for 45 min, and then set on ice. The addition of 1 mL of warm Super Optimal Broth (SOC) media was followed by shaking (250 rpm, 37 °C) for 1 hr. Cells were plated on an LB Amp plate to screen for the desired mutant plasmid. Mutant dsDNA was confirmed by DNA sequencing at the UIUC Core Sequencing Facility. Cells were co-transformed with pTGro7 plasmid containing the GroES-GroEL chaperonin system.

### 4.3. Protein Expression and Purification of CYP2C8*1/*2/*3/R139K/K399R

All CYP2C8 proteins were expressed according to the protocol used in CYP2J2 expression, as previously described [41,44]. The protein concentrations were determined using a UV–vis spectrophotometer (Agilent Technologies, Santa Clara, CA, USA) (ε = 108 mM^−1^·cm^−1^).

### 4.4. Expression of Cytochrome P450 Reductase

Expression of CPR from *Rattus norvegicus* was performed as previously described [41,44].

### 4.5. Assembly of CYP2C8-Nanodiscs

CYP2C8-ND were assembled as previously described [37,44,45] by mixing CYP2C8, membrane scaffold protein (MSP1E3D1), an 80:20 ratio of POPC:POPS lipids, and cholate, followed by detergent removal using Amberlite^®^ beads, and purification by size exclusion chromatography [31,32].

### 4.6. Carbon Monoxide Binding Assay

The heme content of the purified CYP2C8 proteins was analyzed using UV–vis spectroscopy (Agilent Technologies) as previously described [37].

### 4.7. Paclitaxel Metabolism

Samples containing 0.1 μM of CYP2C8-ND (*1/*2/*3/R139K/ K399R) were incubated with CPR (0.3 μM), and PAC (70 μM) in 0.3 mL of 0.1 M potassium phosphate buffer (pH 7.4) for 5 min at 37 °C. NADPH (200 μM) was added and the mixture was incubated for 5, 10, and 20 min at 37 °C, then quenched with equivolume ethyl acetate. Samples were vortexed and thrice-extracted with ethyl acetate, dried under a stream of N_2_ gas, and then resuspended in 180 proof ethanol for LC–MS/MS quantification.

### 4.8. Tandem LC–MS/MS for the Quantification of 6α-Hydroxypaclitaxel

Samples were analyzed with the 5500 QTRAP LC/MS/MS system (Sciex, Framingham, MA, USA) in Metabolomics Lab of Roy J. Carver Biotechnology Center, University of Illinois at Urbana-Champaign. Software Analyst 1.6.2 was used for data acquisition and analysis. The 1200 series HPLC system (Agilent Technologies) includes a degasser, an autosampler, and a binary pump. The LC separation was performed on an Agilent Eclipse XDB-C18 (4.6 × 150 mm, 5 μm) with mobile phase A (0.1% formic acid in water) and mobile phase B (0.1% formic acid in acetontrile). The flow rate was 0.4 mL/min. The linear gradient was as follows: 0–2 min, 95%A; 8–15 min, 5%A; 15.5–22 min, 95%A. The autosampler was set at 15 °C. The injection volume was 5 μL. Mass spectra were acquired under positive electrospray ionization (ESI) with the ion spray voltage at +5000 V. The source temperature was 450 °C. The curtain gas, ion source gas 1, and ion source gas 2 were 32, 50, and 65, respectively. Multiple reaction monitoring (MRM) was used for quantitation: Paclitaxel *m/z* 854.4 → *m/z* 569.2; 6α-hydroxypaclitaxel *m/z* 870.4 → *m/z* 286.2. Internal standard carbamazepine was monitored at *m/z* 237.1 → *m/z* 194.0.

### 4.9. HOOH Measurements

Hydrogen peroxide measurements were made using an Amplex Red Hydrogen Peroxide/Horseradish peroxidase (HRP) Kit (Life Technologies, Waltham, MA, USA) according to the published protocol. Amplex Red combined with HRP reacts with HOOH in a 1:1 stoichiometry producing the red-fluorescent oxidation product, resorufin (A_560nm_). Samples containing 0.1 μM of CYP2C8-ND (*1/*2/*3/R139K/ K399R) were incubated with CPR (0.3 μM) in 0.3 mL of 0.1 M potassium phosphate buffer (pH 7.4), ± paclitaxel (70 μM), for 5 min at 37 °C. NADPH (200 μM) was added and the mixture was incubated for 10, 15, and 20 min at 37 °C, then quenched with equivolume ethyl acetate, vortexed thoroughly, and centrifuged at 3000 rpm at 4 °C for 5 min. The aqueous fraction containing HOOH was extracted and centrifuged at 10,000 rpm at 4 °C for 10 min to remove precipitated protein and lipids. Next, 50 μL of each sample was diluted eight-fold and sixteen-fold and combined with Amplex Red/HRP (10 mM Amplex Red, 10 U/mL HRP in 1× reaction buffer) in a clean, dry 96-well plate. Each sample was analyzed in triplicate. The reactions were incubated at room temperature for 30 min in the dark. The UV A_560nm_ was measured using a microplate reader. Baseline corrected absorbance values of each sample were compared to a standard curve ([HOOH] = 0 to 20 μM).

### 4.10. NADPH Assay

The rate of NADPH (ε = 6.2 108 mM^−1^·cm^−1^ at A_340nm_) consumption by each CYP2C8 variant (0.2 μM) incubated with CPR (0.6 μM) and PAC (70 μM) in 0.1 M potassium phosphate buffer and 200 μM NADPH was determined via UV–vis spectroscopy using a Cary 300 UV–vis spectrometer in kinetics mode (Agilent Technologies), as previously described [41,44].

### 4.11. Stopped-flow Kinetics of Electron Transfer

An Applied Photophysics SX-17 MV Spectrophotometer (Leatherhead UK) was used to monitor the reduction of CYP2C8*1/*2/*3/R139K/K399R, as previously described with the following modifications [41]. Reaction cell 1 containing CYP2C8 (2 μM) in 0.1% cholate, CPR (2 or 6 μM), paclitaxel (70 μM), glucose oxidase (1 U/mL), and glucose (10 mM) dissolved in 100 mM potassium phosphate buffer was kept anaerobic and CO-saturated. Reaction cell 2 containing excess NADPH (1 mM), paclitaxel (70 μM), glucose oxidase (1 U/mL), and glucose (10 mM) dissolved in 100 mM potassium phosphate buffer was also kept anaerobic. The reaction cells were kept at 4 °C until rapid mixing followed by absorbance readings at 37 °C.

### 4.12. Data Analysis of Stopped-Flow Experiments

The reduction of ferric CYP2C8 to a ferrous–CO complex was monitored near A_450nm_ upon mixing the two separate reaction cells in logarithmic mode and analyzed as described previously, with the following changes [41]. All data indicate the average of 3–6 individual reactions fitted using either a monophasic or biphasic exponential equation using OriginPro 2017. The initial decrease in absorbance at 450 nm (A_450nm_) corresponding to the rapid reduction of CPR were not included in these analyses due to spectroscopic noise. *R^2^* values for fits exceeded 0.99 in most cases. The errors reported are SEM.

### 4.13. Reduction Potential

Reduction potential of the CYP2C8 proteins was determined using safranin T as a redox probe as previously described [37]. Samples containing 5 µM of CYP2C8 variant, 20 nM paraquat (methyl viologen), 0.5 µM safarinin T, 10 mM EDTA, 50 µM of a 20% lipid reconstituted system [46], with or without 70 µM paclitaxel were prepared in 0.1 M potassium phosphate buffer, pH 7.4, in glass vials capped with septa. Samples were purged with N_2 (g)_ for 20 min and then loaded into a Coy anaerobic glove box. 0.5 mL of each samples was loaded into UV–invisible plastic cuvettes stopped with a septa. Reduction potential was determined spectroscopically using a Cary 300 UV–vis spectrometer (Agilent Technologies). Cuvettes were equilibrated at 25 °C for each reading. Safranin T was used as the redox indicator to measure the reduction potential of the solution. Oxidation of the protein was monitored at 417 nm (reduction at 408 nm) and compared to the oxidation of safranin T at 535 nm. Reduction of the samples was initially achieved by irradiating samples on ice with time points up to 5 min with a 250 W tungsten lamp. Samples were further reduced by titrating anaerobic dithionite from 8 and 80 mM stocks. Re-oxidation was achieved by titrating anaerobic K_3_[Fe(CN)_6_] from 10 mM stocks. Spectral data were then processed using a MATLAB (R2014a) subroutine and analyzed using the Nernst equation as previously described [37].

## Figures and Tables

**Table 1:** Paclitaxel (PAC) metabolism by CYP2C8 variants. Linear rates of 70 μM PAC metabolism by each CYP2C8 variant to PAC–OH was determined over a 20 min period. Estimates of the catalytic efficiencies were determined using Equation (1) as stated in the text. Error represents the SEM of three experiments.

Variant	Rate (pmol/min/nmol_prot_)	%WT	k_cat_/K_m_ (min^−1^ nM^−1^)	%WT
CYP2C8*1 (WT)	38.8 ± 0.2	100	0.381	100
CYP2C8*2	18.3 ± 3.5	47.2	0.207	54.4
CYP2C8*3	34.0 ± 3.0	88	0.321	84.4
CYP2C8-R139K	103 ± 2	265	1.02	269
CYP2C8-K399R	47.1 ± 4.3	121	0.441	116

**Table 2:** Hydrogen peroxide (HOOH) production per PAC turnover. The amount of HOOH formed at 20 min was divided by the amount of PAC–OH produced at 20 min. These values are compared to WT.

Variant	nmol_HOOH_/pmol_PAC-OH_ (at 20 min)	%WT
CYP2C8*1 (WT)	1.58	100
CYP2C8*2	2.96	187
CYP2C8*3	3.25	206
CYP2C8-R139K	0.46	29.6
CYP2C8-K399R	1.24	78.4

**Table 3:** Reduction potentials. Reduction potentials of the CYP2C8 variants was determined as described in the text. Error represents the SEM of two experiments.

Variant	Reduction Potential (V)
CYP2C8*1 No PAC	−0.283 ± 0.002
CYP2C8*1	−0.279 ± 0.002
CYP2C8*2	−0.297 ± 0.021
CYP2C8*3	−0.281 ± 0.001

**Table 4:** Stopped-flow CO binding kinetics. Fast (k_1_) and slow (k_2_) rates are in units of ms^−1^. Experiments were conducted using a 1:1 and a 1:3 CYP:CPR ratios. Error represents the SEM of 3–6 experiments. Statistical significance compares to WT. ** *p* < 0.01, *** *p* = 0.001, **** *p* < 0.0001.

Variant	1:1 CPR:CYP		3:1 CPR:CYP
	k_1_ (ms^−1^)	k_2_ (ms^−1^)	k_2_ (ms^−1^)
CYP2C8*1	468 ± 21	20.7 ± 0.4	9.70 ± 0.98
CYP2C8*2	---	8.67 ± 0.49 ****	17.4 ± 1.6 ***
CYP2C8*3	---	10.8 ± 1.84 ****	13.7 ± 0.7 ****
CYP2C8-R139K	406 ± 52	18.2 ± 0.65	6.02 ± 0.19 **
CYP2C8-K399R	445 ± 51	19.4 ± 0.99	3.90 ± 0.10 ***

## Supplementary Materials

Supplementary materials can be found at https://www.mdpi.com/1422-0067/20/18/4626/s1.

## Abbreviations

Amp	Ampicillin
AA	Arachidonic acid
Chlr	Chloramphenicol
CYP	Cytochrome P450
CYP2C8	Cytochrome P450 2C8
CYP2C8*3	Cytochrome P450 2C8 R139K/K399R
CYP2C8*2	Cytochrome P450 2C8 I269F
CPR	Cytochrome P450 reductase
δ-ALA	δ-Aminolevulinic acid
EETs	Epoxyeicosatrienoic acids
FET	First electron transfer
HOOH	Hydrogen peroxide
IPTG	Isopropyl β-D-1-thiogalactopyranoside
ND	Nanodisc
POPC	1-palmitoyl-2-oleoyl-sn-glycero-3-phosphocholine
PAC	Paclitaxel
PAC-OH	6α-Hydroxypaclitaxel
POPS	1-Palmitoyl-2-oleoyl-sn-glycero-3-phospho-L-serine
ROS	Reactive oxygen species
