# Supplementary material for: Polymorphisms of CYP2C8 Alter First-Electron Transfer Kinetics and Increase Catalytic Uncoupling
Source: Int J Mol Sci. 2019 Sep 18;20(18):4626. doi: 10.3390/ijms20184626 (PMC6769586; doi:10.3390/ijms20184626)
Supplement: Supplementary file 1 [file ijms-20-04626-s001.pdf]

Figures

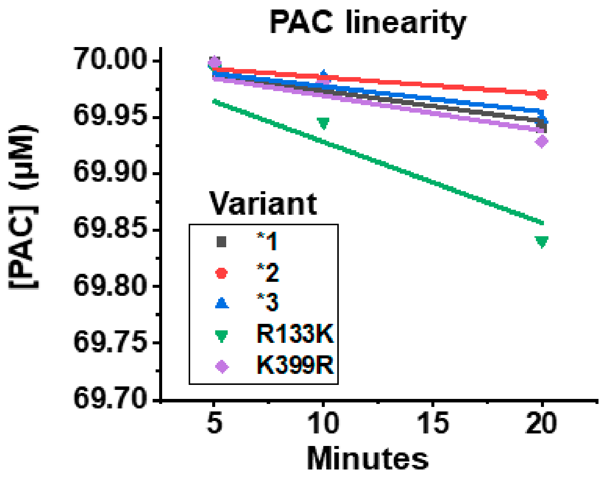

Figure S1. PAC linearity and catalytic efficiency estimates. Concentration of PAC was determined by subtracting the amount of PAC-OH measured from 70 µM of the starting PAC. Catalytic efficiency was estimated using Equation 1 as stated in the text. Data represents the mean of 3 replicates.

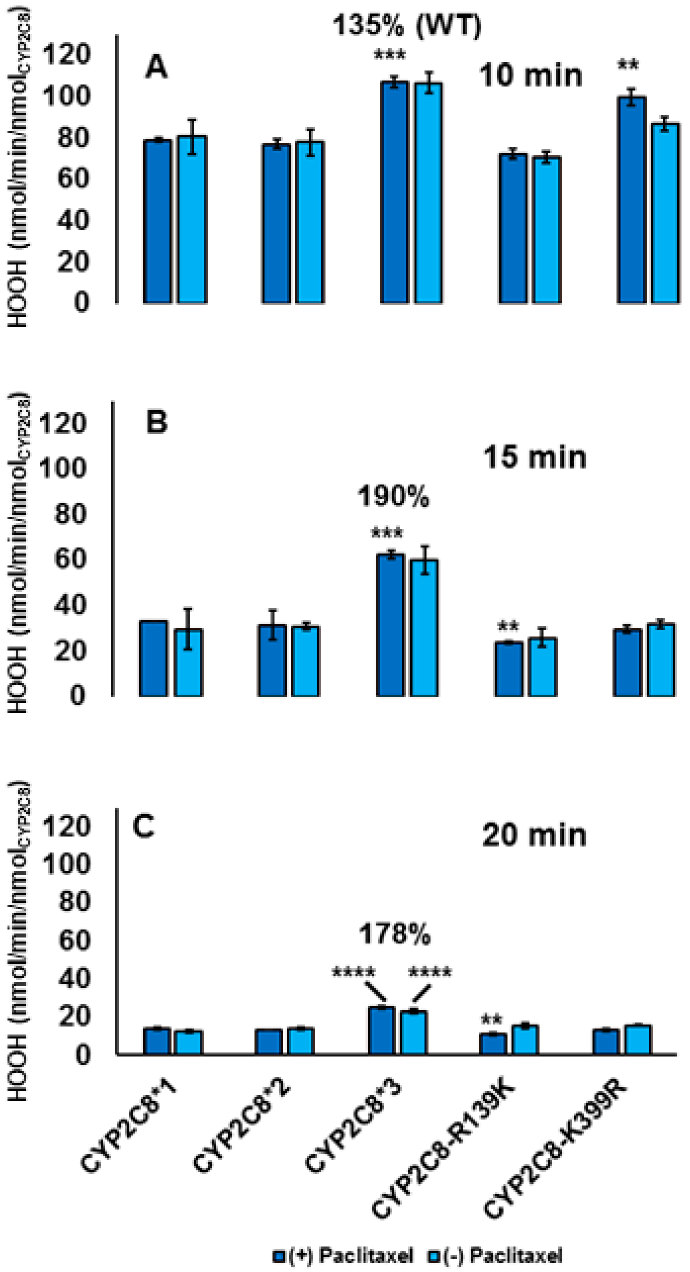

Figure S2. H_2O_2 production rates. The rate of H_2O_2 production by each CYP2C8 variant was measured using an Amplex Red peroxidase kit at (**A**) 10 min, (**B**) 15 min, and (**C**) 20 min reaction times, with and without 70 μM paclitaxel. Error represents the SEM of at least three experiments. Statistic significance was determined by comparing (+) and (-) paclitaxel experiments to their respective WT controls. ** $p < 0.01$; *** $p = 0.0001$; **** $p < 0.0001$.

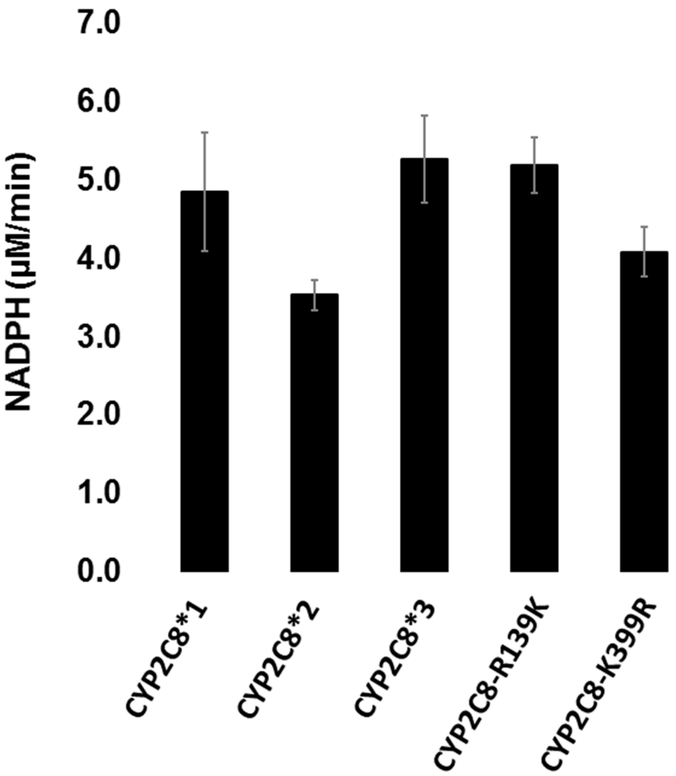

Figure S3. NADPH consumption rates. Rates of NADPH consumption were measured by monitoring the decrease in absorbance at λ = 340 nm as NADPH is converted to $NADP^+$ by CPR. NADPH consumption levels are close to WT (CYP2C8*1) in all variants, with slight, albeit statistically not significant decreases in CYP2C8*2 and CYP2C8-K399R. Error represents the SEM of at least three experiments.

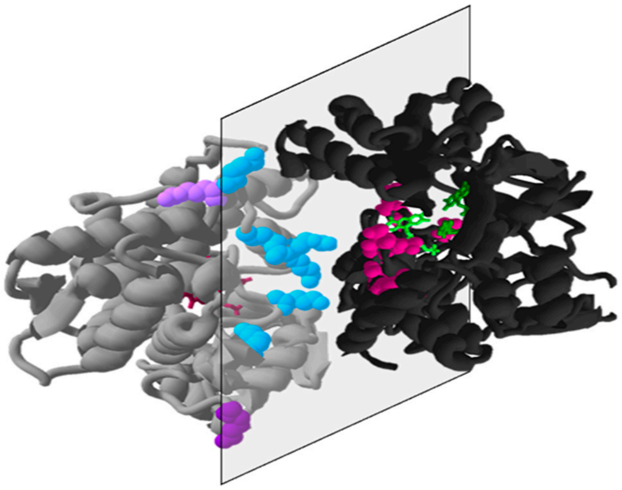

Figure S4. Schematic of CPR-CYP2C8 complex highlighting interface residues and the location of CYP2C8*3 mutations. The amino acid residues involved in CYP-CPR interaction are highlighted based on a previously published model of CYP2B4-CPR, as CYP2C8 and CYP2B4 share high homology. The backbone of CYP2C8 is displayed as grey ribbons and the heme is dark red. CYP2C8*3 polymorphic mutations R139 and K399 are highlighted as light and dark purple spheres, respectively. Positively-charged arginine and lysine residues identified in CYP2B4-CPR binding (K121, R125, R132, K138, K421, K432, R442, Bridges et al. 1998) are displayed on CYP2C8 in blue. The CPR backbone is displayed as black ribbons with its FAD and FMN domains in dark and light green, respectively.

Negatively charged residues implicated in binding to CYPs (E142, D144, D147, D207, D208, D209, E213, E214, D215 by Zhao et al. 1999; Shen/Kasper et al. 1995) are displayed in magenta. Images were generated using PyMOL software.

Tables

Table S1. Mutagenesis primers.

Mutation	Primer Sequence (5′ → 3′)
**I269F**	
Forward	TCAATTGCTCTTCGTTCAAAATGGAGCAGGAAAAGGACAACCAAA
Reverse	TCGAGCGCTCTTCTGAACAGGAAGCAATCGATAAAGTCCCG
**R139K**	
Forward	TTCGCATGCTCTTCAAGAGCATTGAGGACCGTGTTCAAGAGG
Reverse	AAGCTCGCTCTTCCTCTTCTTCCCCATCCCAAAATTCCG
**K399R**	
Forward	TAGTACGCTCTTCCAGAGAATTTCCTAATCCAAATATCTTTGACC
Reverse	GGTTTAGCTCTTCCTCTGTCATCATGTAGCACGGAAGTCAGT

Table S2. Spectral Characterization of CYP2C8 and mutants. Wavelength values were obtained from spectra of substrate-free, oxidized protein samples and Fe(II) CO-bound samples of CYP2C8, *3, R139K, and K399R protein.

Spectral Characteristics of CYP2C8					
	**CYP2C8*1**	**CYP2C8*2**	**CYP2C8*3**	**CYP2C8-R139K**	**CYP2C8-K399R**
Oxidized λ (nm)	416	416	416	420	420
CO-bound λ (nm)	450	451	450	450	450
